# Research on Risk Assessment and Prediction of RMB Internationalization Based on the PCA-SA-BPNN Model

**DOI:** 10.1155/2022/5132718

**Published:** 2022-09-29

**Authors:** Shili Hu, Jie Du

**Affiliations:** School of Economics, Jilin University, Changchun 130000, Jilin, China

## Abstract

This paper combines principal component analysis, a BP neural network, and a simulated annealing algorithm, to construct a PCA-SA-BPNN risk forecast model to evaluate and predict the RMB internationalization risk status of China. First, we analyze the risk of RMB internationalization and its transmission mechanism from the perspective of the economic characteristics of neighboring countries and trading partner countries. Second, we use the FASP index system construction method for reference to construct a forecast index system for macro- and microrisks brought about by RMB internationalization. Then, the weight of each index is determined through index common degree analysis and principal component analysis, and the risk of RMB internationalization is divided. On this basis, the risks of RMB internationalization in China from 2000 to 2019 are divided into four categories. Based on the BP neural network algorithm optimized by the simulated degradation algorithm, the PCA-SA-BPNN model of RMB internationalization risk forecast is constructed. Finally, the validity of the model is verified by experimental verification, and the risk status of RMB internationalization in 2020 is simulated and predicted. The research results show that the risk status of RMB internationalization in 2020 is basically safe, and the risks of RMB internationalization mainly come from macroeconomic growth risks and systemic risks of the financial system.

## 1. Introduction

Dated back to the 1980s when RMB internationalization started to attract academic attention. Yet, due to the low level of China's economic development and the underdeveloped financial market at that time, the discussion was generally carried out within China only. As China's economy enjoyed a long period of high growth, its sovereign currency, the RMB, also experienced increasing international influence, and its internationalization gradually became a magnetic topic both academically and practically. Following the exchange rate system reformation in 2005 and the global financial crisis in 2008, Chinese authorities became aware of the necessity of RMB internationalization should China be to keep its global status both economically and politically. Then in 2009, RMB officially started internationalization, following the traditional path of becoming a regional currency first then to global currency. After years of development especially by founding off-shore financial centers, RMB is now facing severe competition from US dollar, the dominant currency pricing most of the commodities globally. This confrontation forces Chinese authorities to reconsider and look for a new path to expand usage of RMB internationally while averting direct competition from other major currencies. Then the “Belt and Road Initiative” (the “B&R”), raised in 2013, with a full set of trading and financing policies, gives RMB a new channel to achieve internationalization without having much conflict with existing major currencies. The B&R aiming at free trade and capital flow and targeting helping underdeveloped economies along the region is promoting RMB internationalization with the following mechanics: first, economies along the B&R, which can hardly get enough funding from the World Bank Group, can borrow RMB from China to finance their infrastructure; second, during the construction process, it is much more convenient to purchase stuff from China and settle in RMB as the fund borrowed in the first step is in RMB; third, as the fund is borrowed, it will have to be paid back in the future, which means should the economies be avoided being exposed to exchange risks, its best choice would be to put RMB in their foreign reserves and pay it back when the loan matures. Through this mechanism, RMB can gradually serve the function of international trade settlement, international financing, and foreign reserves, and therefore achieving internationalization.

The delicately designed B&R policy system can theoretically promote RMB internationalization. Yet risks to Chinese macroeconomy and financial system may arise due to the specific economic and geopolitical characteristics of the countries along the B&R. Those risks, including hyperinflation, unstable economic growth, unsound jurisdiction, volatile political situation, and nationalism, may affect the inflation of Chinese economy, default risk exposure of Chinese financial system and volatility of Chinese financial markets through unpredictable capital flows. Therefore, research on risk transmission mechanism and forecasting is urgently needed.

Ever since the start of new millennium, RMB internationalization became a hot topic among scholars around the world as China has the strongest growth economically and the largest market in consumption of commodities. Massive research was conducted on the logic, the path, the prospects, and the risks of RMB internationalization, all of which were based on classical theories or empirical evidence from developed economies as the United Kingdom, Germany, Japan, and EU, as a result, those researches were general and can hardly be of practical use. As the B&R was initiated, much attention was paid to the study of its mechanism to promote RMB internationalization, while research on the risk it may bring about is rarely studied.

Before initiation of the B&R, most researches are based on classical scenarios of currency internationalization to study the conditions to meet and the possible paths to follow. Also, there are researches focusing on the risk side with historical data from developed economies that were applied and conclusions were made that China has to further strengthen its economic growth, its capital flow managerial skills, and its exchange rate to evade issues already incurred in Germany and Japan.

Among the researches some scholars believe that the prospects of RMB internationalization is promising if certain conditions are met. Nakagawa thought that free convertibility of RMB would bring fundamental changes to the Asian monetary system. Therefore, East Asian countries should take corresponding measures [1]. Stier et al. believed that China's prudent opening of capital accounts and straining exchange rate fluctuations within a managable range are the first step of RMB internationalization, and it is also a correct choice to take Hong Kong as the experimental center of RMB internationalization [2]. Park believed that a regional cooperation strategy should be further strengthened in the process of RMB internationalization, and an international currency framework should be established in the ASEAN region at the same time that an efficient modern financial system is established in the country [3].

Some scholars hold different views and seem to be cautious about the prospects. Tadokoro believed that even if China's economic growth is impressive, it is impossible for RMB to compete with the US dollar if it is not fully convertible [4]. Subramanian and Kessler collected information on a sample of emerging market economies for empirical research and showed that the RMB is gradually becoming a regional anchor currency, an RMB area has been formed in East Asia, and a more globalized RMB area may be formed in the mid-2030s [5]. Tung et al. applied the principal component analysis method to construct a currency internationalization degree measure composed of two indicators using RMB and 32 other major currencies and found that the degree of RMB internationalization is still low, far behind the four major international currencies [6]. Bowles and Wang pointed out that during the process of RMB internationalization, the international monetary system is also evolving and the US dollar's dominance faces a harsh winter [7]. Eichengreen and Kawai argued that although offshore financial center in Hong Kong played a significant role in the great progress of RMB internationalization, the currency remains highly constrained due to the lack of capital account liberalization [8]. Ito and Kawai investigated the experience of the US dollar, Japanese yen and Deutsche Mark in their own use for international trade settlement from the 1970s to the 1990s, trying to provide lessons for RMB internationalization. They found that for China, to make RMB an international currency, the financial market should be further opened, and per capita income should be increased [9]. It seems that in the field of scholars' research, some believe that the prospect of RMB internationalization is encouraging, while others believe that there are constraints that drag the process.

With regard to researches on the risk of currency internationalization, scholars mostly focus on the risk of the financial system. The IMF and World Bank use financial soundness indicators (FSIs) in their Financial Sector Assessment Program (FSAP) to assess the overall risks and vulnerabilities of member countries' financial systems. The European Central Bank (2010) establishes a comprehensive system of risk forecast indicators. In recent years, many new methods have been innovated in financial risk forecast research in academic circles. For example, Nag and Mitra developed a currency crisis early warning system by using an artificial neural network [10]. Lehar obtained the joint default probability by using the Coupla structure for marginal default risk information and used it to measure systemic risk [11]. Kritzman built a network based on mutual exposure of assets and liabilities and transaction data among financial institutions and simulated risk contagion to calculate systemic risk [12]. Zhang proposed an autoregressive conditional risk model to identify whether a financial crisis occurs according to a time-dependent critical state and give forecast of risks [13]. Frankel and Saravelos selected six indicators, including GDP growth rate, currency depreciation degree, foreign exchange reserves, stock index, and industrial output, as the crisis warning indicator system [14]. Ognjen used Jones, Lauren, and Alexander polynomials for theoretical analysis to study the mechanism of financial crises and crisis contagion and to predict them through polynomials [15].

In terms of the risk forecast, Dobson and Masson believe that RMB internationalization relaxes the supervision of banks' foreign exchange business and completely builds on the basis of commercialization, which will make state-owned banks experience difficulties. Banks' foreign exchange business under the flexible exchange rate also opens the door to financial risk [16]. Ma and Mccauley believes that once China's capital controls are relaxed, RMB internationalization will lead to the failure of the bond market rationing mechanism, deposit and loan interest rate controls, and credit scale control policies [17]. Barry and Masahiro believe that RMB internationalization will pose a challenge to China's monetary policy authorities because the accelerated process of RMB internationalization will inevitably require further opening of the capital account and increase the flexibility of the RMB exchange rate, while excessive capital account liberalization may put China's financial market at risk [18].

Some scholars have revealed the risks existing in the process of RMB internationalization to varying degrees and have found some interesting results. However, the problem is that the existing research is too rough and not concrete enough.

Since proposition of the B&R in 2013, a great deal of research about its impact on RMB internationalization have been conducted. However, the scholars mainly focus on the mechanism of the B&R to promote RMB internationalization and rarely involves discussion about the risks it brings to China's macroeconomy and financial system. In the opinion of some scholars, the B&R is more like a Chinese version of the “Marshall Plan,” and can play an innovative role in promoting RMB internationalization. However, unlike the Marshall Plan, the RMB will inevitably conflict with mainstream international currencies during the process of its internationalization through B&R, and it is difficult to determine what the final effect will be. Shannon, after comparing the B&R and the “Marshall Plan,” believes that both are carriers of rising powers trying to increase their influence in other regions by economic means and to promote the internationalization of their own currencies [19]. Fratzscher and Mehl, after comparing the influence of the RMB, EUR, JPY, and USD in the countries along the B&R by using the exchange rate linkage network model, believe that the influence of RMB on the region was still much lower than that of the other three currencies [20]. Some scholars are also optimistic that the RMB can achieve full internationalization through the implementation of the B&R. Landry and Tang believe that as the US government implements protectionism policies, the availability and attractiveness of the RMB will gradually increase among countries along the Belt and Road, and the international monetary system will be in the process of alienating the US dollar and promoting the internationalization of the RMB [21].

In terms of the risks brought to China's macroeconomy and financial system by RMB internationalization promoted by the B&R, Ito believes that the pricing function of a currency can better reflect the degree of currency internationalization than the settlement function [22]. China's neighboring countries generally use the US dollar as the currency for trade pricing, resulting in that China's economy more affected by the United States even when RMB achieves internationalization along the B&R. Although the above scholars have studied the process and risks of RMB internationalization based on the B&R, they rarely take a look at composition of the risks and mechanisms of forecasting.

Therefore, the existing research mostly focuses on the discussion of the route and the general risk profile and transmission mechanism of RMB internationalization. In regard to the B&R, most of the existing research discusses how RMB internationalization can be promoted through the B&R, while few scholars have studied the risks arising therefrom, let alone research and exploration of the risk forecast mechanism. In consideration of the above, this paper uses the principal component analysis method to establish a corresponding risk forecast indicator on the basis of theoretical discussion on the risk and transmission mechanism of RMB internationalization through the “B&R.”

## 2. Construction of the RMB Internationalization Risk Forecast Index System

### 2.1. Construction of the Forecast Index System

This paper constructs an index system of 19 specific indices at three levels. Since the changes in the selected indices and the direction of risk changes are different, this paper divides the 19 indices by referring to the method of dividing the indices into positive indices, negative indices, and moderate indices in financial analysis. These index data are from the wind database. A positive index means that the larger the index value is, the higher the corresponding risk, such as the unemployment rate and nonperforming loan ratio of the banking system. The negative index means that the smaller the index value is, the higher the corresponding risk, such as the growth rate of total retail sales of consumer goods and the current account growth rate. The moderate index means that the index within a certain range can be regarded as low risk, lower than the lower limit of the range or higher than the upper limit, and the corresponding risk will gradually increase, such as CPI and expected change of RMB exchange rate. The details are shown in Table 1:

This indicator system includes 7 positive indicators, 6 negative indicators, and 6 moderate indicators. Among them, for the moderate range of moderate indicators, this paper adopts the simple empirical assignment method, that is, using the upper and lower limits of indicators in China for a total of 20 years from 2000 to 2019 as a sample and comprehensively considering the median, mean, standard deviation, and mode of the sample to determine the upper and lower limits of moderate indicators. Since 2000, China's economy has made rapid progress in integrating into the world economy. Correspondingly, the impact of world economic fluctuations on China's economy has become increasingly obvious. Therefore, this paper selects indicators since 2000, which can better reflect the feedback of China's economic indicators on external fluctuations. The specific range is shown in Table 2:

### 2.2. Standardization of the Index System

Although the index system constructed above covers various fields, such as macroeconomics, financial systems, and monetary systems, and reflects the risks brought by currency internationalization to a country's economy and finances, there are many problems, such as an excessive number of indices, uneven dimensions of indices and correlations among indices, and it is difficult to construct a forecast index based on this index system. Before data analysis, it is usually necessary to standardize the data and use the standardized data for data analysis. Data standardization is also the indexation of statistical data. Data normalization processing mainly includes two aspects: data co-chemotactic processing and dimensionless processing. The specific steps are as follows:

The positive index is standardized forward. The higher the positive index value, the greater the risk of RMB internationalization, such as the “registered urban unemployment rate.” The standardized value of the data *y*_*ki*_ can be expressed as follows:

(1) $$\begin{array}{c} y_{ki}=\frac{x_{ki}-\underset{1\leq k\leq N}{\text{min}x_{ki}}}{\underset{1\leq k\leq N}{\text{max}x_{ki}}-\underset{1\leq k\leq N}{\text{min}x_{ki}}}. \end{array}$$

The negative index is reversely standardized, and the larger the negative index value is, the smaller the risk of RMB internationalization, such as the “core capital adequacy ratio.” The standardized value of the data *y*_*ki*_ can be expressed as follows:

(2) $$\begin{array}{c} y_{ki}=\frac{\underset{1\leq k\leq N}{\text{max}x_{ki}}-x_{ki}}{\underset{1\leq k\leq N}{\text{max}x_{ki}}-\underset{1\leq i\leq N}{\text{min}x_{ki}}}. \end{array}$$

The moderate index is first subjected to the centralization process, namely, −1 × |*X* − *K*|, where K is the moderate coefficient value, and then the forward normalization process is performed (as shown in Formula (1)).

## 3. PCA-SA-BPNN Model of RMB Internationalization Risk Forecast

### 3.1. Principal Component Analysis (PCA)

In this paper, the principal component analysis method is used to deal with the index system. To fully consider the forecast error caused by the influencing factors related to the original data, principal component analysis was used to complete the feature extraction process. Principal component analysis (PCA) reduces the input data of *n* p-dimensions into m-dimensions, where *m* < *p*. The PCA method is essentially a basis transformation, which makes the transformed data have the maximum variance. After coordinate transformation, the orthogonal axis with high variance is removed to obtain the dimensionally reduced dataset. The steps of dimensionality reduction in principal component analysis are as follows:

Construct the sample matrix with *n* p-dimensional samples:

(3) $$\begin{array}{c} X=\left[\begin{array}{cccc} x_{11} & x_{12} & \cdots & x_{1p}\\ x_{21} & x_{22} & \cdots & x_{2p}\\ \vdots & \vdots & \ddots & \vdots \\ x_{n1} & x_{n2} & \cdots & x_{np} \end{array}\right]. \end{array}$$

Centralize the sample matrix, that is, each sample minus the sample mean: $x_{i}-\bar{x_{i}}$, where

(4) $$\begin{array}{c} \bar{xi}=\frac{1}{n}\sum_{i=0}^{n}x_{i}. \end{array}$$

The sample variance is used as a measure of dispersion:

(5) $$\begin{array}{c} S^{*2}=\frac{1}{n}\sum_{i=1}^{n}{\left(x_{i}-\bar{x_{i}}\right)}^{2}. \end{array}$$

Sample variance along the axis *v*.

Define the vectors *v* ∈ *R*^*ρ*^ and ‖*v*‖=1. Projecting *x*_*i*_ vertically onto the vector *v*, the new coordinates can be expressed as <*x*_*i*_, *v*>. The sample variance in this direction is expressed as follows:

(6) $$\begin{array}{c} \frac{1}{n}\sum_{i=1}^{n}{\left(<x_{i},v>-\sum_{i=1}^{n}<x_{k},v>\right)}^{2}. \end{array}$$

After matrix centralization, the above equation degenerates into

(7) $$\begin{array}{c} \frac{1}{n}\sum_{i=1}^{n}<x_{i}-v{>}^{2}. \end{array}$$

Written in matrix form as follows:

(8) $$\begin{array}{c} \frac{1}{n}{\left\|Xv\right\|}^{2}. \end{array}$$

Maximize the variance in a certain direction and find the direction that maximizes the sample variance based on the sample variance in the above direction *v*:

(9) $$\begin{array}{c} \text{argmax}{\left\|Xv\right\|}^{2},1-{\left\|v\right\|}^{2}=0. \end{array}$$

Using the Lagrange multiplier method, the maximization problem with constraints is transformed into a maximization problem without constraints:

(10) $$\begin{array}{c} \text{argmax}{\left\|Xv\right\|}^{2}+\mu \left(1-{\left\|v\right\|}^{2}\right). \end{array}$$

Let Γ(*v*, *μ*)=‖*Xv*‖^2^+*μ*(1 − ‖*v*‖^2^). Taking the derivative with respect to the direction *v* and setting the derivative equal to 0, we have

(11) $$\begin{array}{c} \frac{1}{n}X^{T}Xv=\frac{\mu }{n}v. \end{array}$$

The obtained first direction *v* is an eigenvector of the covariance matrix of the sample *V*=1/*nX*^*T*^*X*, and *μ*/*n* is the corresponding eigenvalue. In the same way, using the Lagrange multiplier method and adding the constraint <*v*, *v*_1_>, the remaining principal directions are obtained.

### 3.2. BP Neural Network Model for Simulated Annealing Algorithm Optimization (SA-BPNN)

**Figure 1:**
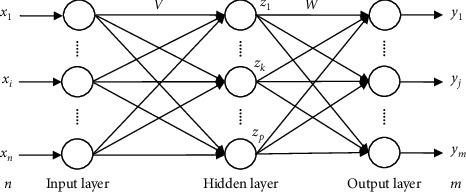
Structure diagram of the BP neural network.

The BP neural network model is a multilayer feed-forward neural network model trained according to the error backpropagation algorithm, which consists of two processes: forward propagation of the data stream and backpropagation of the error signal. The forward propagation direction is “input layer ⟶ hidden layer ⟶ output layer,” and the neuron state of each layer only affects the neurons in the next layer. If the output layer does not obtain the desired result, then the backpropagation process of the error signal is used, and the two processes are carried out alternately. The error function gradient descent strategy is implemented in the weight vector space, the network error function is minimized by dynamically iterating a set of weight vectors, and information extraction and training are completed, as shown in Figure 1.

Assuming that the input layer of the network model structure has *n* nodes, the hidden layer has *q* nodes, and the output layer has *m* nodes, the weight between the input layer and the hidden layer is *v*_*ki*_, and the weight between the hidden layer and the input layer is *w*_*jk*_. The transfer function of the hidden layer is *f*_1_(·), the transfer function of the input layer is *f*_2_(·), and the output of the hidden layer node is

(12) $$\begin{array}{c} z_{k}=f_{1}\left(\sum_{i=0}^{n}v_{ki}x_{i}\right)\;\left(k=1,2,3,\ldots ,q\right). \end{array}$$

The node input of the output layer is

(13) $$\begin{array}{c} y_{j}=f_{2}\left(\sum_{i=0}^{n}w_{jk}z_{k}\right)\;\left(j=1,2,3,\cdots ,m\right). \end{array}$$

The above process reflects the forward propagation process of the BP neural network data flow. When the output result is inconsistent with the expected output result, it turns to the backpropagation of the error information. Assuming that *p* training samples are input, denoted by *x*_1_, *x*_2_,…, *x*_*p*_, the output of the *p*th sample after input into the network is *y*_*j*_^*p*^(*j*=1,2,3*|* …, *m*). The error conduction function is set as a squared error function; that is, the error of the *p*th sample *E*_*p*_ can be expressed as follows:

(14) $$\begin{array}{c} E_{p}=\frac{1}{2}\sum_{j=1}^{m}{\left(t_{j}^{p}-y_{j}^{p}\right)}^{2}. \end{array}$$

where *t*_*j*_^*p*^ is the expected output result and the total error for all samples is expressed as follows:

(15) $$\begin{array}{c} E=\frac{1}{2}\sum_{p=1}^{p}\sum_{j=1}^{m}\left(t_{j}^{p}-y_{j}^{p}\right), \end{array}$$

(16) $$\begin{array}{c} \Delta w_{jk}=-\eta \frac{\partial E}{\partial w_{jk}}=-\eta \frac{\partial }{\partial w_{jk}}\left(\sum_{p=1}^{p}E_{p}\right), \end{array}$$

(17) $$\begin{array}{c} \mu_{yj}=-\frac{\partial E}{\partial S_{j}}=-\frac{\partial E_{p}}{\partial y_{j}}\cdot \frac{\partial y_{j}}{\partial S_{j}} \end{array}$$

(18) $$\begin{array}{c} \frac{\partial E_{p}}{\partial w_{jk}}=\frac{\partial E_{p}}{\partial S_{j}}\cdot \frac{\partial S_{j}}{\partial w_{jk}}=-\mu_{yj}z_{k} \end{array}$$

(19) $$\begin{array}{c} \Delta w_{jk}=\sum_{p=1}^{p}\sum_{j=1}^{m}\eta \left(t_{j}^{p}-y_{j}^{p}\right)f_{2}^{\prime }\left(S_{j}\right)z_{k}, \end{array}$$

(20) $$\begin{array}{c} \Delta v_{jk}=\sum_{p=1}^{p}\sum_{j=1}^{m}\eta \left(t_{j}^{p}-y_{j}^{p}\right)w_{jk}f_{1}^{\prime }\left(S_{k}\right)\cdot x_{i}. \end{array}$$

However, the standard BP neural network has problems such as slow convergence, and the network itself easily falls into the local minimum. The standard BP neural network uses the method based on gradient descent to search for the optimal solution. Obviously, if the error function has only one local minimum, then this local minimum is deemed the global minimum; then, if the error function has multiple local minimums, it cannot be guaranteed that the solution is the global minimum. For the latter case, parameter optimization falls into a local minimum.

To avoid this problem, this paper uses the simulated annealing technique. Simulated annealing accepts a result worse than the current solution with a certain probability at each step, which helps it escape the local minimum. In the iterative process of each step, the probability of accepting a “suboptimal solution” gradually decreases over time, thus ensuring the stability of the algorithm.

In this paper, the idea of probabilistic acceptance of new solutions in the simulated annealing (SA) algorithm is introduced into the BP neural network to find a model parameter initialization scheme to promote the accuracy of the forecasts.

Simulated annealing first initializes the current temperature *T*=*T*_0_, randomly generates an initial solution *w* and generates a new solution *ρ* through random disturbance. Then, it calculates and judges the increment Δ*f* of the new solution and updates the state according to the following state transition rules:

(21) $$\begin{array}{c} P\left(w⟶p\right)=\left\{\begin{array}{c} e^{f\left(w\right)-f\left(\rho \right)/T_{0},}\\ 1. \end{array}\right. \end{array}$$

**Figure 2:**
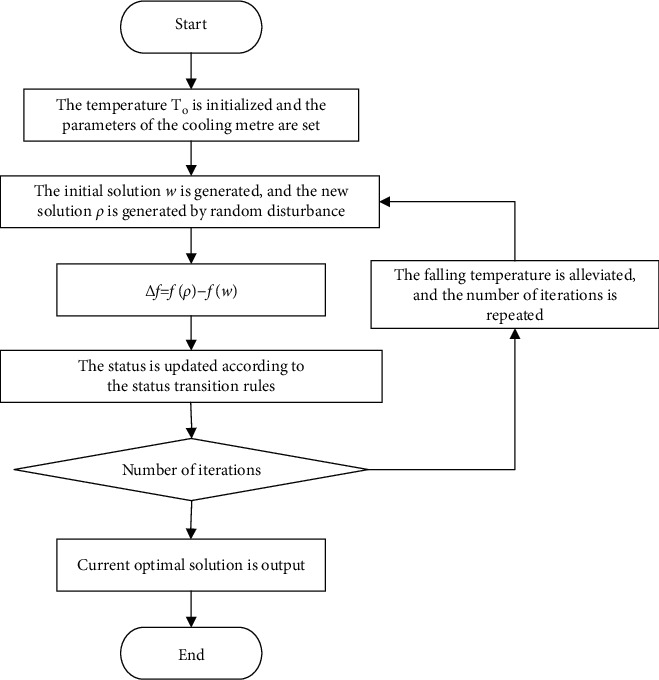
Simulated annealing optimization flow.

When the state no longer shows transition during the annealing process, the current state is output, and the cooling function with the same step size is selected to slowly cool down to ensure that the cooling process reaches thermal equilibrium at each temperature and check whether iteration termination conditions are met. Otherwise, the initial solution is regenerated after decay *T* according to decay function *T*(*n*+1)=*K* × *T*(*n*). The above annealing and cooling process is repeated until the optimal solution is output. The simulated annealing optimization flow is shown in Figure 2.

The simulated annealing algorithm is used to find the optimal solution of the initial parameters of the BP neural network, and a neural network forecast model (SA-BP neural network) optimized by the simulated annealing algorithm is constructed for forecast. The forecast steps are as follows:

  Step 1: The load data are randomly sorted, and the test and training data are divided according to the ratio of 1 : 3.
  Step 2: The sample input and output data are normalized.
  Step 3: The BP neural network parameters are set, and a three-layer BP neural network model, with 3 nodes in the input layer, 9 nodes in the hidden layer, and 1 node in the output layer, is adopted.
  Step 4: The optimal solution according to the simulated annealing algorithm flow is output.
  Step 5: The optimal solution is used as the initial weight threshold of the BP neural network, the features extracted by the principal component analysis are used as the input of the BP neural network, and the SA-BP neural network forecast model is constructed.
  Step 7: The normalized training data are used to train the SA-BP neural network forecast model and make forecasts.
  Step 8: The output results are denormalized, and the error between the predicted value and the actual value is calculated.
  Step 9: Whether the error or the number of iterations meets the set conditions is determined, and the forecast result is output if the conditions are met; otherwise, the result is iteratively optimized until the conditions are met.

**Figure 3:**
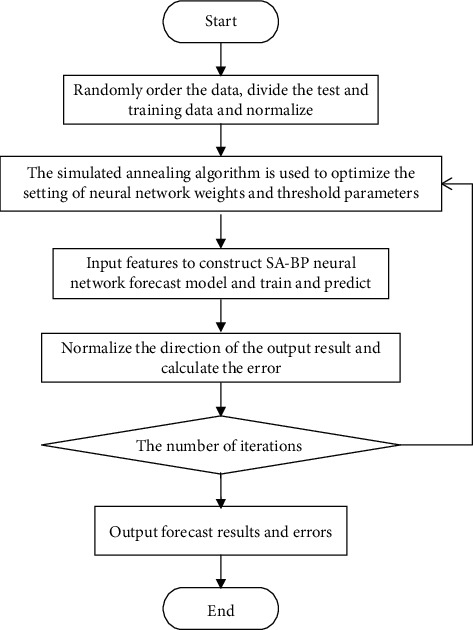
Output flow of the SA-BP neural network forecast model.

The improved forecast flow is shown in Figure 3.

## 4. Empirical Analysis

### 4.1. Principal Component Analysis and Division of RMB Internationalization Risk Forecast Status

#### 4.1.1. Common Degree Analysis and Principal Component Extraction

The initial common degree of each risk forecast index is set to 1.  Table 3 shows that the common degree values corresponding to all indices are higher than 0.6, indicating a strong correlation between indices and factors, and factors can effectively extract information. With the assurance that the indicators extract the information of the research items, this paper continues to analyze the relationship between indices and factors.

Principal component extraction should follow the following three principles: the eigenvalue is greater than 1, the cumulative variance contribution rate is greater than 80%, and pass the steep slope test. As shown in Table 4, the extraction of principal components and the amount of information extracted from principal components were analyzed. It can be seen from the above table that a total of 5 principal components were extracted by principal component analysis, and the eigenvalues were all greater than 1. The variance explanation rates of these five principal components were 52.685%, 9.661%, 8.396%, 7.777%, and 5.330%, respectively, and the cumulative variance explanation rate was 83.849%. In addition, a total of five principal components were extracted in this analysis, and their corresponding weighted variance explanation rate, that is, weights, was 62.83%, 11.52%, 10.01%, 9.28%, and 6.36% (52.685/83.849 = 62.83%; 9.661/83.849 = 11.52%; 8.396/83.849 = 10.01%; 7.777/83.849 = 9.28%; 5.330/83.849 = 6.36%).

**Figure 4:**
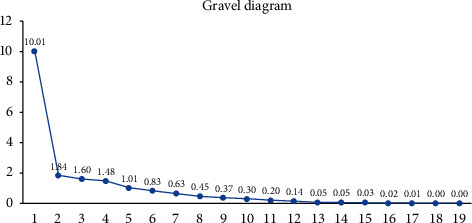
Gravel diagram.

The gravel diagram is used to assist in judging the number of extracted principal components. When the stitches are suddenly changed from steep to smooth, the number of principal components corresponding to the changes is the number of extracted principal components for reference. As shown in Figure 4, after the fifth principal component, the gravel diagram completely stabilizes, which shows that it is reasonable to divide the risk of RMB internationalization into five principal components in this paper.

#### 4.1.2. Indicator Weight Determination

Principal component analysis can perform weight calculations by using load coefficient information.

The calculation process is divided into three steps. First, the linear combination coefficient matrix is calculated, which means that the load coefficient is divided by the square root of the corresponding characteristic root. Second, the comprehensive score coefficient is determined, that is, the product of the cumulative linear combination coefficient is multiplied by the variance explanation rate and divided by the cumulative variance explanation rate, which means that linear combination coefficients multiply the variance explanation rate, accumulate the products, and then divide by the cumulative variance explained rate. Finally, the weight is calculated, and the comprehensive score coefficient is normalized to obtain the weight value of each indicator. The final indicators and weights are shown in the table below.

From the perspective of risk weights of major categories (see Table 5), the risks brought by RMB internationalization under the “B&R” are mainly reflected in macroeconomic growth risks and currency risks, with weights of 32.74% and 30.59%, respectively. This result is reasonable. The volatile economic growth and unpredictable inflation will lead to unstable capital flows into and out of China, forcing the People's Bank of China to increase or decrease target rates; also, as the Central Asian countries are important suppliers of minerals to China, their domestic inflation will be reflected in the price of raw minerals, thus transmitting inflation into Chinese economy. On the other side, the volatile economic and geopolitical conditions in economies along the B&R will lead to volatile demand of RMB. For instance, if a nationalist party becomes the ruling party of a country, it will naturally show hostility to trade with China, leading to local business to abandon using RMB as settlement currency and the existing RMB will flow back to China, forcing RMB to depreciate.

The systemic risk of the financial system comes second, with a weight of 25.9%, and the impact of inflation risk is smaller, with only 10.78%. Macroeconomic growth risk weight and currency risk weight are the highest mainly because the two types of risks contain the largest number of indicators; the risk weight of the financial system is significantly lower than the first two, mainly because the weight of the average price-earnings ratio of the CSI 300 Index is significantly lower, which shows that this indicator cannot well reflect the risks brought by currency internationalization to China's capital market and indirectly reflects that China's capital market is still a relatively closed market. From the perspective of a single indicator, the nonperforming loan ratio of the banking system, the foreign debt service ratio, and the registered urban unemployment rate ranked in the top three in terms of weight, reflecting that currency internationalization brings risks to the issuing country, and it is prominently reflected in the unemployment rate being superior in the macroeconomic field. The financial system will be mainly reflected in the nonperforming ratio of the banking system, and the currency field will be mainly reflected in the ratio of foreign debt service. The weights of major categories of risks and the ranking of the weights of single indicators can provide a reference for subsequent analysis of the causes of forecast indicator alarms and the formulation of corresponding policies.

#### 4.1.3. Index Threshold Determination and Risk Status Division

The variance contribution rates of the five principal factors were weighted and averaged to obtain the risk value reflecting the risk of RMB internationalization, and the “extreme value - mean estimation method” was used to define the critical value of the risk state. The variance contribution rates of the five principal factors are weighted and averaged to obtain the risk value reflecting the risk profile of RMB internationalization, and the “extreme value − mean estimation method” was applied to define the critical value of the risk status. We set different thresholds for indicators at all levels according to the order from local to global, to achieve correct classification of risk levels. When we get the quantitative valuation of a certain level of risk, we can grade the risk, and further to practice the prewarning system. Since the parent-level indicators are obtained through the weighted average method of sublevel parallel indicators, we first need to classify the risk level of the indicators at the end level (Level 4) of the evaluation index system. After trend identity and dimensionless standardization, the indicators of the fourth level change in the same direction with the systematic risk, and the average value is 0 and the standard deviation is 1. According to this distribution feature, the results of our designed risk threshold and risk level are as follows: The interval [0, (max − min)/4] was defined as the safe state. The interval ((max − min)/4, (max − min)/2] was defined as the basically safe state. The interval ((max − min)/2, 3(max − min)/4] was defined as the alert state. The interval (3(max − min)/4, max] was defined as the dangerous state. The specific results are shown in Table 6.

Considering that the output function of the neural network model only recognizes variables of “0/1,” vectors A (0001), B (0010), C (0100), and D (1000) were used to represent the risk status of RMB internationalization over the years. The results of RMB internationalization risk status from 2000 to 2019 are shown in Table 7:

### 4.2. Learning, Training, and Simulation of the PCA-SA-BPNN RMB Internationalization Risk Forecast Model

**Figure 5:**
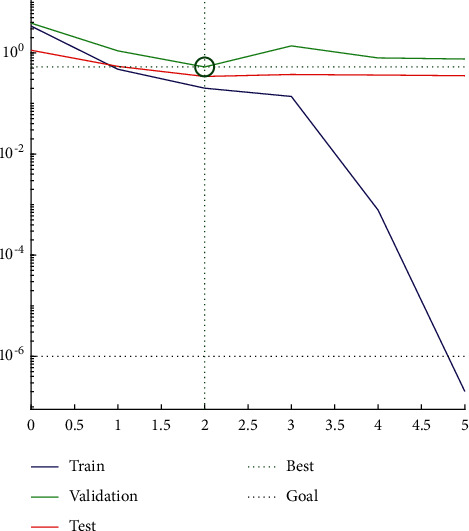
Training convergence process diagram of the optimized BP neural network.

**Figure 6:**
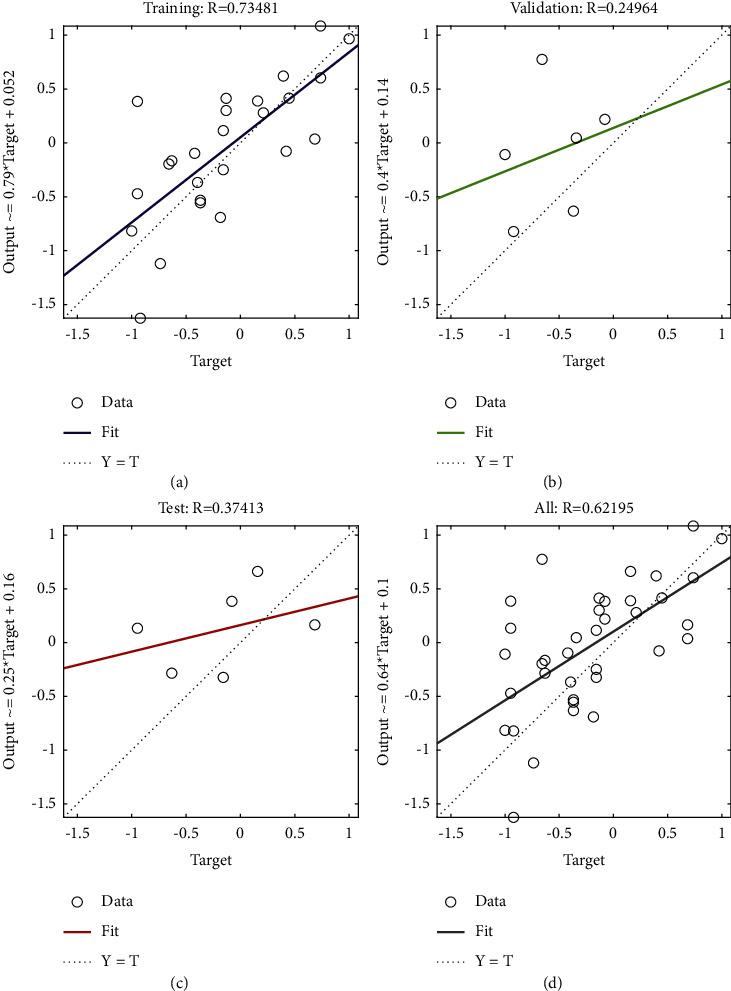
Goodness of fit of the BP neural network model after optimization. (a) Training: *R* = 0.73481; (b) validation: *R* = 0.24964; (c) test: *R* = 0.37413; (d) all: *R* = 0.62195.

According to the constructed PCA-SA-BPNN RMB internationalization risk forecast model, historical data and information were used for network learning and training. That is, the samples from 2000 to 2016 were used as the input neurons of the training set, and the data and information of the current year were used to predict the risk state of RMB internationalization in the next year. The training process was mainly carried out through the Sim function in MATLAB for simulation learning and training. The network learning process and convergence results are shown in Figure 5. According to the training results, after network learning and training on historical data, the error between the output layer of the neural network and the training target was only 5.28637 × 10^−4^, the goodness of fit of the model training set was 0.73481, and the goodness of fit of the test set was 0.24964 (as is shown in Figure 6). The PCA-SA-BPNN model can better reflect the mapping relationship between risk forecast indices and the risk states of RMB internationalization after network training, so a systemic financial risk forecast model can be constructed based on the PCA-SA-BPNN algorithm.

### 4.3. Forecast Results of the PCA-SA-BPNN RMB Internationalization Risk Forecast Model

**Figure 7:**
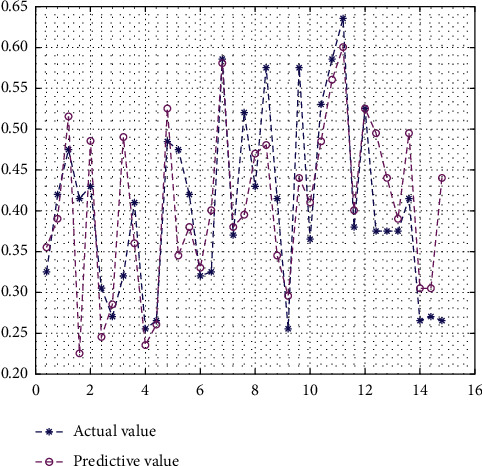
Comparison of the actual values and the forecasted values.

Before using the PCA-SA-BPNN RMB internationalization risk forecast model, it is necessary to test the forecast produced by the model using data from 2017, 2018, and 2019 as the test set samples. The trained PCA-SA-BPNN RMB internationalization risk forecast model and the Sim simulation function in MATLAB were used to predict the risk states of the test set samples, and the risk states were compared with those divided by principal component analysis to judge the accuracy of the forecast of the risk forecast model. The test results are shown in Table 8. The test results show that the forecast results of the PCA-SA-BPNN model of the test set samples were basically consistent with the vector corresponding to the risk states obtained by the principal component analysis, as shown in Figure 7. This indicates that the RMB internationalization risk forecast model constructed based on the PCA-SA-BPNN algorithm can accurately predict the risk states.

Using the data from 2019 as the input layer neuron input model of the PCA-SA-BPNN forecast model, the risk status of RMB internationalization in 2020 was predicted. According to the forecast results of the PCA-SA-BPNN RMB internationalization risk forecast model for RMB internationalization risk status in 2020, the Sim simulation output results of RMB internationalization risk status in 2020 were expressed as a vector (0.0000, 0.0031 0.8232 0.0042). This is basically consistent with the vector expression (0 0 1 0) of the risk state B determined by the principal component analysis. The result of the PCA-SA-BPNN RMB internationalization risk forecast model for the RMB internationalization risk state in 2020 basically predicts a safe state. Further combined with the current development state of RMB internationalization at this stage, the highest scoring principal factors in the sample of the forecast set are the first principal factor and the second principal factor. The risks of RMB internationalization in China are mainly focused on the macroeconomic growth risk and systemic risk of the financial system at the present stage.

### 4.4. Backtesting of the Forecast Indicator

**Figure 8:**
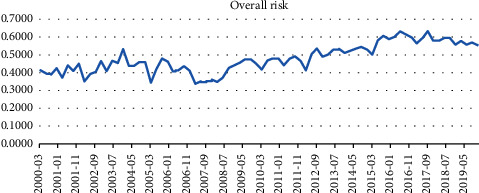
Overall risk evolution diagram of the macroeconomic and financial systems.

To test whether the established forecast indicator is effective, this paper backtests the forecast indicators with 80 quarters as samples from January 2000 to December 2019 for a total of 20 years. As shown in Figure 8, the overall risk began to enter the low-risk region in June 2008 and gradually increased thereafter.

**Figure 9:**
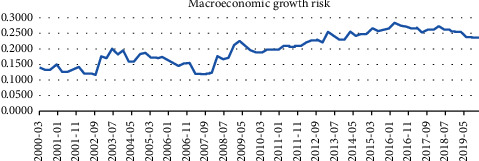
Macroeconomic growth risk.

From a preliminary point of view, China's macroeconomic growth risk has been on the rise since the global economy fell into recession due to the financial crisis in June 2008. In combination with the “Four trillion Plan” economic stimulus plan launched by the Chinese government in 2009, it seems that China's policies have no effect on the macroeconomy. However, against the background that all major economies in the world fell into recession, the central banks of the United States, Japan, and Europe have adopted several rounds of QE, and the Bank of Japan has maintained negative interest rates even now. Despite the slowdown in economic growth in China, the Chinese government's timely policy stimulus has ensured that the macroeconomy has not fallen into recession like other economies (as shown in Figure 9), and since September 2016, China's macroeconomic growth risk has gradually declined, showing that China's economic recovery has been faster than other major economies. At the same time, the quality of economic growth and the ability to resist risks are also improving. It has been confirmed by backtesting that the forecast index can issue an alarm before risk events occur. If the government responds properly and in a timely manner, it can effectively reduce the impact of risk events on the macroeconomic and financial systems.

## 5. Conclusion

While the B&R provides a new path for the internationalization of the RMB, it also poses new risks to China's macroeconomic and financial systems. Considering the unstable economic growth, high external debt, and geopolitical complexities of countries along the Belt and Road, promoting the internationalization of the RMB through the B&R will make the RMB the currency for investment, financing, and trade settlement. Even foreign exchange reserves could pose new risks to China's macroeconomic and financial systems. Such risks will become increasingly prominent as China's investments in countries along the Belt and Road continue to increase.

Based on the scientific validation of the established indicators, this paper uses a BP artificial neural network model to make a preliminary forecast analysis of the financial risk in RMB internationalization. Based on the estimation results of the BP artificial neural network model, the possibility of a financial crisis in China is low, and most of the indicators perform well. Nevertheless, as the B&R continues to carry out, this paper gives rise to a new way for policymakers to quantitatively identify indicators that forecast alarming macroeconomic risks and evaluate the scale of those risks arising from the process of RMB internationalization. Then corresponding actions targeting the risky indicators can be taken to eliminate the risks. For instance, the impact of exchange rate fluctuations, the international environment, and asset price fluctuations may be more significant. Furthermore, although the forecast indicator system constructed in this paper can obtain good estimation results when verifying and warning, the internationalization of the RMB involves a wide range of issues and is complicated. The current forecast of financial risks in RMB internationalization is a forward-looking approach. In other words, the study of financial risk forecast in the process of RMB internationalization needs to be further evaluated and improved.

## Figures and Tables

**Table 1:** Macroeconomic and financial indicator system.

Primary classification	Secondary classification	Tertiary classification	Correlation	Mark
Macroeconomic growth risks	Aggregate	Actual GDP growth rate	Moderate indicator	I1
		Growth rate of per capita disposable income of residents	Negative indicator	I2
		Registered urban unemployment rate	Positive indicator	I3
	Structure	Fixed asset investment growth rate	Negative indicator	I4
		Growth rate of total volume of retail sales	Negative indicator	I5
		Expected changes in RMB exchange rate	Moderate indicator	I6

Systemic risks in the financial system	Micro risk	Core capital adequacy ratio	Negative indicator	I7
		Cumulative foreign exchange exposure ratio	Positive indicator	I8
	Macro risk	Nonperforming loan ratio of the banking system	Positive indicator	I9
		CSI 300 index average price-earnings ratio	Moderate indicator	I10
		Growth rate of financial account balance	Negative indicator	I11

Inflation risk		Consumer price index (CPI)	Moderate indicator	I12
		M2/GDP	Moderate indicator	I13

Currency risk	CA	Current account balance growth rate	Negative indicator	I14
	KA	Short-term capital balance/financial account balance	Positive indicator	I15
	Sovereign debt risk	Fiscal deficit ratio	Moderate indicator	I16
		National debt burden ratio	Positive indicator	I17
		Foreign debt service ratio	Positive indicator	I18
		Short-term foreign debt/foreign exchange reserve balance	Positive indicator	I19

**Table 2:** Range table of moderate indicators.

Name of indicator	Lower limit	Upper limit
Real GDP growth (annualized)	6.7%	7.1%
Expected changes in RMB exchange rate	6.5	6.8
CSI 300 index average price-earnings ratio	11	17
Consumer price index (CPI)	1.5%	3.0%
M2/GDP	4.98	7.56
Fiscal deficit ratio	3%	5%

**Table 3:** Common degree of each indicator.

Indicator	Initial common degree	Common degree after extraction
I_1_	1.000	0.951
I_2_	1.000	0.791
I_3_	1.000	0.891
I_4_	1.000	0.986
I_5_	1.000	0.924
I_6_	1.000	0.782
I_7_	1.000	0.874
I_8_	1.000	0.892
I_9_	1.000	0.979
I_10_	1.000	0.624
I_11_	1.000	0.777
I_12_	1.000	0.826
I_13_	1.000	0.87
I_14_	1.000	0.826
I_15_	1.000	0.58
I_16_	1.000	0.641
I_17_	1.000	0.806
I_18_	1.000	0.966
I_19_	1.000	0.945

**Table 4:** Explanation degree of principal component variance.

Principal component	Eigenvalue	Variance explanation rate (%)	Cumulative variance explanation rate (%)
1	10.01	52.685	52.685
2	1.836	9.661	62.346
3	1.595	8.396	70.742
4	1.478	7.777	78.519
5	1.013	5.33	83.849
6	0.829	4.365	88.214
7	0.626	3.295	91.509
8	0.449	2.363	93.872
9	0.365	1.922	95.794
10	0.295	1.553	97.347
11	0.198	1.045	98.392
12	0.145	0.761	99.153
13	0.05	0.262	99.414
14	0.048	0.251	99.666
15	0.029	0.151	99.817
16	0.017	0.09	99.907
17	0.011	0.059	99.966
18	0.004	0.021	99.987
19	0.003	0.013	100

**Table 5:** Weight of each indicator.

Primary classification	Indicator	Mark	Weight (%)
Macroeconomic growth risks	Actual GDP growth rate	I_1_	5.87
	Growth rate of per capita disposable income of residents	I_2_	5.31
	Registered urban unemployment rate	I_3_	5.98
	Fixed asset investment growth rate	I_4_	5.60
	Growth rate of total volume of retail sales	I_5_	5.73
	Expected changes in RMB exchange rate	I_6_	4.25

Systemic risks in the financial system	Core capital adequacy ratio	I_7_	5.41
	Cumulative foreign exchange exposure ratio	I_8_	5.70
	Nonperforming loan ratio of the banking system	I_9_	6.08
	CSI 300 index average price-earnings ratio	I_10_	3.68
	Growth rate of financial account balance	I_11_	5.03

Inflation risk	Consumer price index (CPI)	I_12_	5.36
	M2/GDP	I_13_	5.42

Currency risk	Current account balance growth rate	I_14_	3.86
	Short-term capital balance/financial account balance	I_15_	4.83
	Fiscal deficit ratio	I_16_	4.85
	National debt burden ratio	I_17_	5.20
	Foreign debt service ratio	I_18_	6.02
	Short-term foreign debt/foreign exchange reserve balance	I_19_	5.83

**Table 6:** Indicator system threshold.

		Forecast level			
		No risk	Low risk	Moderate risk	High risk
Risk level	Overall risk	(0.3364, 0.4102]	(0.4102, 0.4840]	(0.4840, 0.5578]	(0.5578, 0.6316]
	Macroeconomic growth risk	(0.1161, 0.1576]	(0.1576, 0.1991]	(0.1991, 0.2405]	(0.2405, 0.2820]
	Systemic risk in the financial system	(0, 0.0331]	(0.0331, 0.0661]	(0.0661, 0.0992]	(0.0992, 0.1322]
	Inflation risk	(0.0388, 0.0541]	(0.0541, 0.0694]	(0.0694, 0.0848]	(0.0848, 0.1001]
	Currency risk	(0.0362, 0.0848]	(0.0848, 0.1335]	(0.1335, 0.1821]	(0.1821, 0.2308]

**Table 7:** Division of the forecast status of RMB internationalization risk.

Year	Risk status	Year	Risk status
2000	C (0100)	2010	B (0100)
2001	C (0100)	2011	B (0100)
2002	C (0100)	2012	B (0100)
2003	C (0100)	2013	A (0100)
2004	C (0100)	2014	A (0100)
2005	B (0100)	2015	B (0100)
2006	B (0100)	2016	B (0100)
2007	C (0100)	2017	B (0100)
2008	D (0100)	2018	B (0100)
2009	B (0100)	2019	C (0100)

**Table 8:** Test results of the PCA-SA-BPNN RMB internationalization risk forecast model.

Year	Model forecast value	Corresponding alert states	Corresponding vectors of principal component analysis
2017	0.4576	B (0100)	B (0100)
2018	0.4724	B (0100)	B (0100)
2019	0.4254	B (0100)	C (0100)

## Data Availability

The data used to support the findings of this study were supplied by SHILI HU under license and so cannot be made freely available. Requests for access to these data should be made to SHILI HU(E-mail address:Hsl123@zcst.edu.cn).
